# SARS-COV-2 viroporins activate the NLRP3-inflammasome by the mitochondrial permeability transition pore

**DOI:** 10.3389/fimmu.2023.1064293

**Published:** 2023-02-20

**Authors:** Joseph W. Guarnieri, Alessia Angelin, Deborah G. Murdock, Patrick Schaefer, Prasanth Portluri, Timothy Lie, Jessica Huang, Douglas C. Wallace

**Affiliations:** ^1^ Center for Mitochondrial and Epigenomic Medicine, Division of Human Genetics, The Children’s Hospital of Philadelphia, Philadelphia, PA, United States; ^2^ Department of biology, University of Pennsylvania, Philadelphia, PA, United States; ^3^ Department of Pediatrics, Division of Human Genetics, Perelman School of Medicine, University of Pennsylvania, Philadelphia, PA, United States

**Keywords:** COVID-19, NLRP3-inflammasome, viroporin, mitochondrial permeability transition pore, mitochondria in innate immune responses

## Abstract

**Background:**

Compared to healthy controls, severe COVID19 patients display increased levels of activated NLRP3-inflammasome (NLRP3-I) and interleukin (IL)-1β. SARS-CoV-2 encodes viroporin proteins E and Orf3a(2-E+2-3a) with homologs to SARS-CoV-1, 1-E+1-3a, which elevate NLRP3-I activation; by an unknown mechanism. Thus, we investigated how 2-E+2-3a activates the NLRP3-I to better understand the pathophysiology of severe COVID-19.

**Methods:**

We generated a polycistronic expression-vector co-expressing 2-E+2-3a from a single transcript. To elucidate how 2-E+2-3a activates the NLRP3-I, we reconstituted the NLRP3-I in 293T cells and used THP1-derived macrophages to monitor the secretion of mature IL-1β. Mitochondrial physiology was assessed using fluorescent microscopy and plate reader assays, and the release of mitochondrial DNA (mtDNA) was detected from cytosolic-enriched fractions using Real-Time PCR.

**Results:**

Expression of 2-E+2-3a in 293T cells increased cytosolic Ca++ and elevated mitochondrial Ca++, taken up through the MCUi11-sensitive mitochondrial calcium uniporter. Increased mitochondrial Ca++ stimulated NADH, mitochondrial reactive oxygen species (mROS) production and the release of mtDNA into the cytosol. Expression of 2-E+2-3a in NLRP3-I reconstituted 293T cells and THP1-derived macrophages displayed increased secretion of IL-1β. Increasing mitochondrial antioxidant defenses via treatment with MnTBAP or genetic expression of mCAT abolished 2-E+2-3a elevation of mROS, cytosolic mtDNA levels, and secretion of NLRP3-activated-IL-1β. The 2-E+2-3a-induced release of mtDNA and the secretion of NLRP3-activated-IL-1β were absent in cells lacking mtDNA and blocked in cells treated with the mitochondrial-permeability-pore(mtPTP)-specific inhibitor NIM811.

**Conclusion:**

Our findings revealed that mROS activates the release of mitochondrial DNA via the NIM811-sensitive mitochondrial-permeability-pore(mtPTP), activating the inflammasome. Hence, interventions targeting mROS and the mtPTP may mitigate the severity of COVID-19 cytokine storms.

## Graphical Abstract

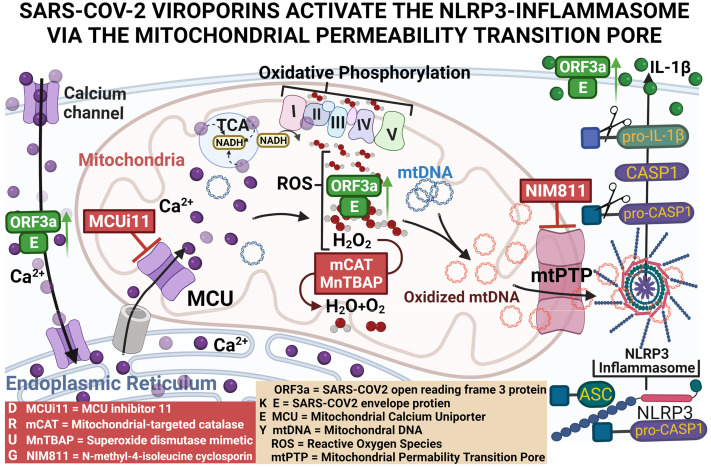

## Introduction

Approximately 609 million cases of COVID-19 have been reported globally, resulting in over 6.5 million deaths (1). COVID-19 is caused by SARS-CoV-2 whose genome structure encodes a polyprotein that is cleaved into 16 non-structural proteins as well as the structural proteins S (Spike), E (Envelope), M (Membrane), and N (Nucleocapsid), plus seven open reading frames (Orfs) 3a, 6, 7a/b, 8, 9b, and 10. Severe COVID-19 manifests as pneumonia, acute respiratory distress syndrome, respiratory failure, and cytokine storm resulting in multiple organ failure (2–4). The cytokine storm results from the elaboration of pro-inflammatory cytokines such as IL-1β (5–7).

The production of mature IL-1β requires the activation of the mitochondrially-bound NLRP3-inflammasome (NLRP3-I), which encompasses the NLR family pyrin domain containing 3 (NLRP3) receptor; the adaptor molecule apoptosis-associated speck-like protein containing a caspase activation and recruitment domain (ASC); and the pro-IL-1β-converting enzyme pro-caspase-1 (CASP1). Upon activation, the NLRP3-I triggers the proteolytic cleavage of pro-caspase-1 (pro-CASP1), and CASP1 cleaves pro-IL-1β and pro-IL-18 to generate IL-1β and IL-18 which are secreted from the cell (8). Autopsy samples from severe COVID-19 patients display increased NLRP3-I activation in lung tissues and peripheral blood mononuclear cells (2), and monocytes isolated from severe COVID-19 patients have increased levels of activated NLRP3-I and IL-1β (4). Thus, understanding the mechanism by which SARS-CoV-2 activates the NLRP3-I is imperative for understanding the pathophysiology of severe COVID-19.

Recently, mitochondrial dysfunction has been shown to activate the innate immune system *via* mROS production and oxidation of the mitochondrial DNA (mtDNA) during replication. mtDNA replication is induced by the expression of the rate-limiting enzyme cytosine monophosphate kinase 2 (CMPK2). The oxidized mtDNA (Ox-mtDNA) is released from the mitochondrion to bind and activate the NLRP3-I (9, 10). While the mechanism by which SARS-CoV-2 activates the NLRP3-I is unknown, expression of the SARS-CoV-1/2 viroporins have been associated with activation of NLRP3-I (11–15) and are known to be membrane ion channels (16, 17).

SARS-CoV-2 encodes two viroporins E (2-E) and ORF3a (2-3a), with homologues to SARS-CoV-1 proteins (18). SARS-CoV-1/2 E and 3a viroporins localize to the endoplasmic reticulum (ER), Golgi apparatus, and plasma membrane (18) where they increase the permeability to cations such as Ca^++^ (19, 20). For SARS-CoV-1, the 1-E and 1-3a have been shown to activate the NLRP3-I in human monocyte-derived macrophages (11, 13, 15). In LPS-primed macrophages, co-expression of 1-E plus 1-3a resulted in higher levels of IL-1β secretion than either viroporin alone (11). 1-E has been reported to activate NLRP3-I through disrupting Ca^++^ homeostasis in cells (12, 14), and activation of the NLRP3-I and secretion of IL-1β by 1-E and 1-3a is mitigated by treatment with the mROS scavenger MitoQ (11). However, the mechanism by which SARS-CoV-2 activates the NLRP3-I has yet to be elucidated.

We hypothesized that expression of 2-E plus 2-3a results in increased Ca^++^ flux into the cytosol where it is taken up by the mitochondrion through the mitochondrial Ca^++^ uniporter (MCU). Within the mitochondrion, the Ca^++^ activates the pyruvate and α-ketoglutarate dehydrogenases to generate excessive NADH (21). The increased NADH overloads the mitochondrial electron transport chain producing increased mROS. The mROS oxidizes the mtDNA, and the Ox-mtDNA is released through the mtPTP to bind to the NLRP3 inflammasome. This activates caspase-1 to cleave pro-IL-1β and pro-IL-18 resulting in the secretion of active IL-1β and IL-18 (10, 22). Our current results support this scenario, thus placing mitochondrial function at the nexus between SARS-CoV-2 infection and the cytokine storm.

## Methods

### Cells, infections, and reagents

293T & THP1 cells were obtained from the American Type Culture Collection (ATCC). Cells were grown at 37°C with an atmosphere of 98% humidity and 5% CO_2_. 293T cells were maintained in Dulbecco’s modified Eagle’s medium + GlutaMAX™ supplement with pyruvate (GIBCO), 1% non-essential amino acids (SIGMA), and 10% fetal bovine serum (FBS) (Takara Bio). 293T-ρ^0^ were generate as previously described in (23). 293T-ρ^0^ cells were maintained in the same media used to maintain 293T cells with the addition of 100 ug/mL of Uridine (SIGMA). THP1 cells were grown in RPMI 1640 Medium (GIBCO) supplemented with 10% FBS (Takara Bio). 293T and 293T-ρ^0^ cells were infected with lenti viruses (MOI 4) as previously described (24). THP1 cells were infected with lenti viruses (MOI 8) with the addition of 10 μg/ml polybrene (VectorBuilder) and spin-inoculated at 700×g for 25min.

### Plasmids, viral vectors, THP1 stable-transformants

#### Plasmid vectors

To express the components of the NLRP3-inflammasome (NLFP3-I), we utilized four plasmids expressing mouse NLRP3 (pcDNA3-N-Flag-NLRP3, Addgene plasmid # 75127), ASC (pcDNA3-N-Flag-ASC1, Addgene plasmid # 75134), CASP1 (pcDNA3-N-Flag-Caspase-1, Addgene plasmid # 75128) and pro-IL-1B (pCMV-pro-Il1b-C-Flag, Addgene plasmid # 75131). The use and construction of the NLRP3-I expression plasmids were previously described (25).

The plasmid vector used to express mitochondrial-targeted catalase (mCAT) and its respective control vector were p-mCAT (VectorBuilder ID VB170403-1078nzg) and p-EV (VectorBuilder ID VB210726-1273jte). The p-mCAT transgene cassette was transcribed from the 212 nucleotide elongation factor α1 “short” (EFS) promoter. The EFS promoter transcribes a polycistronic transcript encoding EGFP (enhanced green fluorescent protein), a self-cleaving 2A peptide site, followed by mCAT cDNA, and terminated by a simian virus 40 (SV40) late polyA sequence. p-EV is identical to the p-mCAT construct, except lacking the EGFP and mCAT sequences.

#### Lentiviral vectors

The lentiviral vector used to co-express 2-E+2-3a was LV-E3a (Vectorbuilder ID VB210112-1153ufz) and its respective control vector LV-EV (Vectorbuilder ID VB210112-1153ufz). The LV-E3a vector contains the *cytomegalovirus* (CMV) promoter, the 2-E+2-3a viroporins obtained from Gordon et al., 2020 (18) separated by a 2A peptide site, and terminated by a SV40 late polyA sequence cloned into the LV-EV vector. The viroporin sequences deduced from the protein sequences were modified by adding an ATG codon 5’ and three N-terminal FLAG-tags added to the 3’ end of each viral protein. LV-EV is an empty vector.

The lentiviral vector expressing our mCAT and its respective control vector were LV-mCAT (VectorBuilder 210909-1242kdf) and LV-EV (VectorBuilder 900122-0484ubz). The LV-mCAT vector includes the EFS promoter, EGFP, 2A peptide site, mCAT, and SV40 late polyA sequence. The LV-EVcontrol vector lacks the EGFP and mCAT sequences.

#### THP1 mCAT stable-transformants

THP1 cells were transduced with LV-mCAT or empty vector and selected with puromycin. Expression of stable-transformants of mCAT were validated by EGFP fluorescence.

## Method details

### Cell staining

293T cells were plated at a density of 45 × 10^3^ in 0.2% gelatin-coated (ScienCell) 96-well glass-bottom plates with high-performance #1.5 mm cover glass (Cellvis). Twenty-four hours post-plating, sub-confluent monolayers of 293T cells were transduced with LV-EV or LV-E3a. Twenty-four hours post-transduction, cells were washed two times with phosphate-buffered saline (PBS), then stained. For determination of mROS levels, cells were co-stained with 3 μM MitoSOX™ Red (MitoSOX, mitochondrial superoxide indicator) and 50 nM MitoTracker™ Deep Red FM (MTDR) for 30 min at 37°C. For assaying mROS levels after treatment with Thapsigargin (TG) using the plate reader, cells were stained with 3 μM MitoSOX for 30 min at 37°C.To quantify mROS after staining, cells were washed three times in PBS, maintained in FluoroBrite™ DMEM (GIBCO) supplemented with 12.5 mM HEPES (SIGMA) and 1% non-essential amino acids (SIGMA), and the fluorescence measured.

To determine cytosolic Ca^++^ levels, cells were washed three times with Tyrode’s Salts (Sigma-Aldrich), stained for 40 min with 2 μM Fura Red™, acetoxymethyl ester (AM), cell-permeant (Fura-Red) in 0.02% pluronic F127 (Pluronic^®^ F-127) detergent. To determine mitochondrial Ca^++^ levels, cells were washed three times with Tyrode’s Salts, stained for 40 min with 10 μM Rhod-2, AM, cell-permeant (Rhod2) and 50 nM MTDR in 0.02% pluronic F127. After staining cells were washed three times, maintained in Tyrode’s Salts, and the florescence measured. To determine mitochondrial Ca^++^ levels after treating with TG, cells were stained for 40 min with 10 μM Rhod2, washed three times, maintained in Tyrode’s Salts, and the florescence measured.

### SpectraMax plate reader assay

#### Measurement of mROS and cytosolic and mitochondrial Ca^++^ levels

After staining cells (see “Cell Staining”), mean fluorescence was assessed using the *SpectraMax® Paradigm® Multi-mode Detection Platform*, equipped with *a* Tunable Wavelength (TUNE) Detection Cartridge (Molecular Devices). mROS was quantified by MitoSOX fluorescence (ex:540 nm, em:590 nm) and MTDR (ex:633 nm, em:680 nm) and MitoSOX/MTDR calculated. Rhod2 fluorescence for mitochondrial Ca*^++^* level (ex:540 nm, em:590 nm) and MTDR (ex:633 nm, em:680 nm), and Rhod2/MTDR calculated. Fura-Red fluorescence was measured for cytosolic bound-Ca^++^ level using ex:405 nm, em:637 nm and unbound-Ca*^++^* level using ex:514 nm, em:672 nm. The ratio of bound-Ca^++^/unbound-Ca^++^ was calculated.

#### Measurement of mitochondrial Ca^++^ and mROS after treating with TG

After staining cells with Rhod2 or MitoSOX (“Cell Staining”), cells were treated for 10 min with or without 10 μM mitochondria channel uniporter inhibitor 11 (MCUi11), or dimethylsulfoxide (DMSO) as a negative control. Mitochondrial Ca^++^ determined from Rhod2 was measured using ex:540 nm, em:590 nm and mROS from MitoSOX using ex:540 nm, em:590 nm. Cells were then treated with 2.5 μM TG and changes in mitochondrial Ca^++^ or mROS recorded every 15 seconds for 180 seconds. Relative change in mitochondrial Ca^++^ was calculated by dividing the average change in Rhod2 fluorescence after treatment with TG by Rhod2 fluorescence before treatment with TG. Relative change in mROS levels were calculated by dividing the average change in MitoSOX fluorescence after treatment with TG by MitoSOX fluorescence before treatment with TG.

### Confocal microscopy

Live-cell fluorescence imaging was performed using a Zeiss 710 LSM confocal microscope with an environmental chamber maintained at 37°C and 5% CO2. Laser lines used: diode 405 nm, Argon 514 nm, HeNe lasers 543 nm and 633 nm excitation wavelengths. Fluorescence was analyzed using ImageJ. Fluorescence intensities of stained cells were normalized to the unstained negative cells. A minimum of 60 images was taken for each condition across at least three independent experiments.

### Fluorescence lifetime imaging microscopy (FLIM) of NADH autofluorescence

Fluorescence lifetime imaging microscopy (FLIM) of NADH autofluorescence was performed according to Schaefer et al. (26). Using FLIM, NADH was measured on a Zeiss LSM 710 Laser Scanning Microscope equipped with a pulsed, two-photon titanium-sapphire laser (80 MHz, 100 fs pulse width). NADH was excited at 730 nm and emission was recorded through a 460/60 nm bandpass filter using the hybrid detector HPM-100-40, mounted on the NDD port of the LSM710. Time-correlated single photon counting (TCSPC) was performed with a temporal resolution of 256 time channels within a pulse period of 12.5 ns, resulting in FLIM images of 512 × 512 pixel. The final measurement settings were 60 sec collection time; ≈ 15 μsec pixel dwell time; 135 × 135 μm^2^ scanning area using a Plan-Apochromat 63x/1.40 Oil DIC M27 lens. SPCM 9.8 was used to record the data, which were subsequently analyzed using SPCImage 8.0 by fitting (WLS method) of a biexponential decay with lifetime components fixed to 400 ps and 2500 ps for free and protein-bound NADH, respectively. Final analysis settings were square binning of 2, peak threshold adapted to background, and shift fixed at a pixel with clear NADH signal. The mean NADH lifetime (t_mean_) was calculated and is depicted in false-color coding with red indicating a shorter and blue a longer NADH lifetime. For subcellular analysis, region of interests (ROIs) of similar size were drawn for five nuclei across each image. For the mitochondria-rich region, the peak threshold was increased to remove nuclear and cytosolic NADH signal, averaging only the t_mean_ of the mitochondrial NADH.

### Catalase assay

Catalase activity was assessed through cleavage of H_2_O_2_ in 293T cell lysates collected twenty-four hours post-transfection with p-mCAT or p-EV, using a Catalysis Activity Kit (Abcam, ab83464).

### Western blot analysis

Twenty-four hours post-transduction, cells were washed with cold PBS and then lysed with 2.5% n-Dodecyl-B-D-Maltoside in 20 mM HEPES (pH 7.4), 50 mM β-glycerophosphate, 2 mM EGTA, 10% (v/v) glycerol, and 0.01% Bromophenol blue. Lysates were electrophoresed on 4 to 12%, Bis-Tris gels (Invitrogen) SDS-polyacrylamide NuPAGE™ gels. Gels were transferred to a nitrocellulose membrane by the iBlot Gel Transfer System (Invitrogen), membranes blocked for one hour in 5% nonfat milk in 150 mM NaCl and 25 mM Tris-buffered saline in 0.1% Tween^®^ 20 Detergent (TBST) then incubated overnight at 4°C with shaking. The primary antibody was diluted 1:1000 in TBST. Membranes were then washed three times with TBST and incubated with Alexa Fluor-conjugated secondary antibodies for one hour at room temperature. Protein levels were quantified using the Odyssey imaging system (LiCOR Biosciences). GAPDH as a loading control.

### Detection of secreted IL-1β in 293T cells with reconstituted NLRP3-I and THP1 macrophages

293T cells were plated at a density of 100 × 10^3^ in 24-well plates. Twenty-four hours post-plating sub-confluent monolayers of 293T cells were infected with LV-EV or LV-E3a. Six hours post-infection cells were co-transfected using the TransIT-X2^®^ Dynamic Delivery System (Mirus Bio) with the plasmids encoding the components of the NLRP3-I (25), followed by p-mCAT or p-EV transduction. Twenty-four hours post-transfection, cells were washed two times with PBS, then cultured with or without the addition of 50 uM MnTBAP. Cell lysates and culture supernatants were collected twelve hours post-treatment and centrifuged to remove cell debris. Supernatant IL-1β was quantified by ELISA (Abcam, ab197742).

THP-1 cells were plated at a density of 100 × 10^3^ in 96-well plates. THP1 cells were infected with LV-EV or LV-E3a. Six hours post-infection, THP1 cells were differentiated into macrophages with 50 ng/ml Phorbol 12-myristate 13-acetate (PMA) overnight. After differentiation, cells were washed two times with PBS, and fresh media was added with the addition of 100 μM MnTBAP, 5 μM MCC950, or 10 μM N-methyl-4-isoleucine-cyclosporin (NIM811). Nine hours post-treatment with MnTBAP or MCC950 or one-hour post-treatment with NIM811, THP1 macrophages were stimulated with 100 ng/ml Lipopolysaccharides (LPS) and 2.5 μM of nigericin for nine hours. Supernatants were collected, centrifuged, and the amount of IL-1β in the supernatants measured by ELISA (Abcam, ab46052). THP1 cells stably expressing LV-mCAT or control vector, were infected with LV-EV or LV-E3a, and 6 hours post-infection the THP1 cells were differentiated into macrophages, and supernatant IL-1β quantified *via* ELISA.

### Detection of mtDNA from cytosolic-enriched fractions in 293T cells

Twenty-four hours post-plating, sub-confluent monolayers of 293T cells were infected with LV-EV or LV-E3a. Six hours post-infection, cells were transfected with the plasmids encoding p-mCAT or p-EV, or twenty-four hours post-infection cells were treated with or without the addition of 50 uM MnTBAP or 10 uM NIM811. Twenty-four hours post-transfection or ten hours post-treatment, cells were washed with PBS, then dissociated from the plate with Trypsin. The dissociated cells were then pelleted *via* centrifugation and lysed with hypertonic buffer consisting of 20 mM Tris-HCl (pH 7.4), 10 mM NaCl, and 3 mM MgCl2 for fifteen minutes on ice, then treated with Non-Ionic Detergent P-40 (NP-40) 0.05% (v/v) and vortexed at max speed. Cell lysates were centrifuged for 15 minutes at 1,000 g, and the resulting supernatants were centrifuged for 1 hour at 200,000 g to isolate a cytosolic enriched fraction. After centrifugation, protein concentrations were determined from the resulting supernatants by Bradford assay using the Bio-Rad protein assay (Bio-Rad, Hercules, CA). DNA was isolated from equal amounts of protein using the DNA Clean & Concentrator-5 (Zymo Research, 11-303). Levels of mitochondrial DNA in the cytosolic enriched fraction were then determined *via* TaqMan Real-Time PCR assays to detect mitochondrial NADH dehydrogenase subunit 5 (*MT-ND5*) using the TaqMan probe (*MT-ND5* Hs02596878_g1).

### Statistical and reproducibility

For quantitative analyses, a minimum of 3 biological replicates were analyzed. Western blot data are from the respective experiment, processed in parallel, and represent at least three independent experiments. One-way ANOVA was performed for statistical differences between three or more groups, followed by a *post hoc* Tukey’s HSD test to test for statistical differences. For studies that require a quantitative evaluation between two groups, statistical significance was determined using unpaired two-tail Student’s t-test. All data bar graphs, data are reported as mean ± standard error of the mean (SEM). All statistical analysis was done on GraphPad Prism 9.01. For student’s t-test * = p < value 0.05, ** = p < value 0.01, *** = p < value 0.001, **** = p < value 0.0001).

## Results

### Expression of 2-E+2-3a increases Ca*^++^* leakage into the cytosol, elevates mitochondrial Ca*^++^* levels, and increases mROS production

A major unanswered question is how 2-E and 2-3a activate the NLRP3-I. We hypothesized that expression of 2-E and 2-3a results in increased Ca^++^ flux into the cytosol where it is taken up by the mitochondrion through the MCU. Within the mitochondrion, the Ca^++^ activates the pyruvate and α-ketoglutarate dehydrogenases to generate excessive NADH. The increased NADH overloads the electron transport chain producing increased mROS. The mROS oxidizes the mtDNA which is released through the mtPTP to bind to the NLRP3-I. This activates caspase 1 to cleave pro-IL-1β and pro-IL-18 resulting in the secretion of active IL-1β and IL-18 (10, 22).

**Figure 1:**
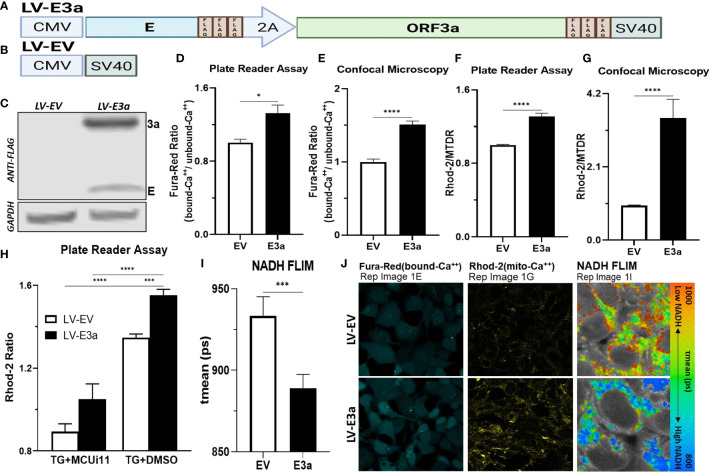
Expression of 2-E+2-3a induces mROS production by elevating mitochondrial Ca*^++^* levels. **(A, B)** Schematics of our LV-EV and LV-E3a vectors. **(C)** 293T cells were transduced with LV-EV or LV-E3a, and samples analyzed by immunoblot with a mouse monoclonal antibody against FLAG-Tag, to detect the FLAG-tagged 2-E+2-3a viroporins. GAPDH was a loading control. **(D, E)** 24 hrs post-transduction cells were stained with Fura-Red to measure cytosolic Ca^++^ levels, **(F, G)** and with Rhod2 & Mitotracker Deep Red (MTDR) to measure mitochondrial Ca^++^ levels**. (E, G, J)** data from confocal microscopy or **(D, F)** data from plate reader assays. **(H)** 24 hrs post-transduction with LV-EV or LV-E3a 293T cells were treated with or without 10 μM MCUi11, and then 2.5 μM TG and stained with Rhod2 to measure mitochondrial Ca^++^ levels by plate reader assays. **(I)** Cells, 24 hrs post-transduction with LV-EV or LV-E3a, were analyzed for mean mitochondrial NADH lifetime (t_mean_) indicating (NAD^+^/NADH ratio) using FLIM. **(J)** Representative images of Fura-Red, Rhod2-stained, and NADH lifetime imaged cells. Scale bar = 30 μm. Error bars represent SEM from 3 independent experiments; statistically significant data is indicated with asterisks (*).

To test this hypothesis, we constructed a polycistronic expression vector combining 2-E+2-3a (LV-E3a) to determine how these viroporins function synergistically to engage the NLRP3-I (**Figures 1A, B**). In this vector the 2-E+2-3a sequences were separated by the self-cleaving 2A peptide site (27) to allow co-expression from a single transcript. The expression of 2-E+2-3a in LV-E3a transduced 293T cells was confirmed by Western blot (**Figure 1C**). We then demonstrated that LV-E3a transduced 293T cells experience increased cytosolic Ca^++^ with Fura-Red by plate reader assay (**Figures 1D, J**) and confocal microscopy (**Figure 1E**). Additionally, we demonstrated that LV-E3a transduced 293T cells experience increased mitochondrial Ca++ with Rhod2 by plate reader assay (**Figures 1F, J** **)** and confocal microscopy (**Figure 1G**). The LV-E3a transduced 293T cells had elevated levels of both cytosolic and mitochondrial Ca^++^.

Treating with thapsigargin (TG) triggers Ca^++^ flux into the cytosol resulting in increased mitochondrial Ca^++^ uptake (28, 29). Treatment with the mitochondrial calcium uniporter inhibitor 11 (MCUi11) blocks Ca^++^ entry into the mitochondrion (30, 31). Treatment with MCUi11 abolished mitochondrial calcium uptake in LV-E3a transduced cells (**Figure 1H**) following TG treatment. Thus, expression of 2-E+2-3a in 293T cells results in increased Ca^++^ flux into the cytosol resulting in an elevation of cytosolic Ca^++^. The cytosolic Ca^++^ is then taken up by the mitochondrial calcium uniporter resulting in elevated mitochondrial Ca^++^ (**Figure 1H**).

Elevated mitochondrial Ca^++^ activates the tricarboxylic acid cycle dehydrogenases generating excess NADH (21). Expression of 2-E+2-3a in 293T cells resulted in increased mitochondrial NADH levels detected *via* NADH fluorescence lifetime imaging (FLIM) (NAD^+^/NADH) mean (ps) (**Figures 1I, J**).

**Figure 2:**
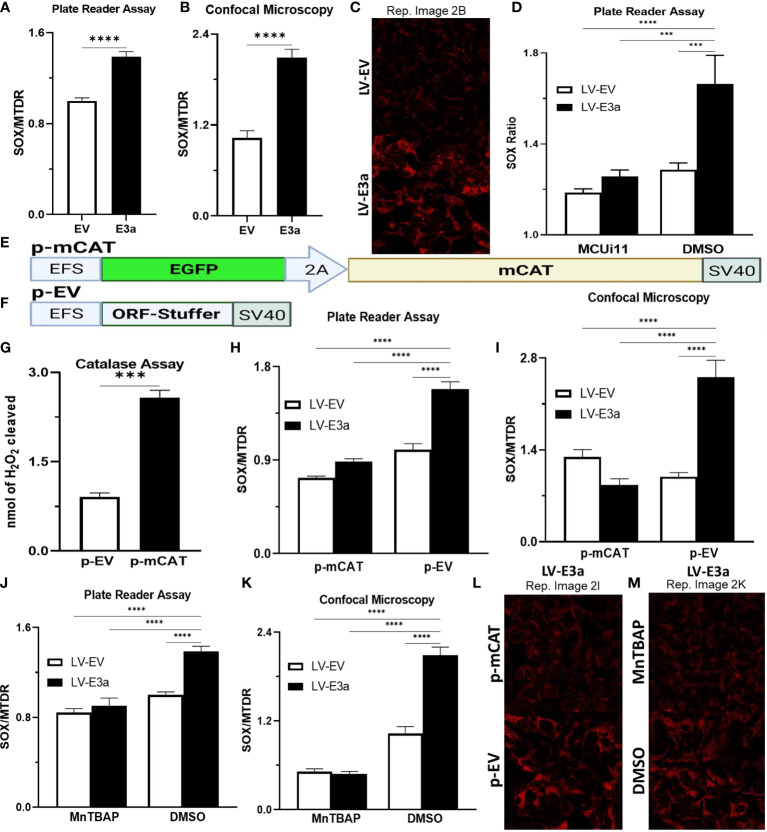
Expression of mCAT or treatment with the mROS scavenger MnTBAP antioxidant defenses blocks 2-E+2-3a induced mROS. **(A–C)** 24 hrs post-transduction cells were stained with MitoSOX and MTDR to measure mROS levels *via* **(A)** data from plate reader assays or **(B, C)** data from confocal microscopy. **(D)** 24 hrs post-transduction with LV-EV or LV-E3a 293T, cells were treated with or without 10 μM MCUi11, and then 2.5 μM TG and stained with MitoSOX to measure mROS levels by plate reader assays. **(E, F)** Schematics of p- p-mCAT and EV vectors. **(G)** Catalase assay through cleavage of H_2_O_2_ in 293T cell lysates collected 24 hrs post-transfection with p-EV or p-mCAT. **(H-M)** 24 hrs post-transfection levels of mROS assessed using MitoSOX and MTDR fluorescence 293T cells transduced with LV-EV or LV-E3a and transfected **(H, I, L)** with p-mCAT or its respective control p-EV or **(J, K, M)** cultured in the presence or absence of 50 μM MnTBAP, DMSO used as a negative control. MTDR fluorescence was used to normalize for mitochondrial content with mROS expressed as the ratio of MitoSOX/MTDR, by **(H, J)** plate reader assays or **(I, K, L, M)** confocal microscopy. **(C, L, M)** Representative images of MitoSOX-stained cells. Scale bar = 30 μm. Error bars represent SEM from 3 independent experiments; statistically significant data is indicated with asterisks (*).

Excessive NADH levels can overload the electron transport chain producing increased mROS. The increased NDAH in 2-E+2-3a expressing 293T cells was associated with increased mROS production detected by staining transduced cells with MitoSOX, which detects mitochondrial superoxide anion production (**Figures 2A–C**). To determine if the increased mROS was due to the entry of Ca^++^ into the mitochondrion, we treated the cells with MCUi11 which blocked the increased mROS production (**Figure 2D**). As predicted MCUi11 blocked the increased mROS production. Hence the increased mROS production in 2-E+2-3a cells was dependent on elevated mitochondrial Ca^++^.

To confirm that the 2-E+2-3a induced ROS production was mROS, we transfected the 2-E+2-3a expressing 293T cells with a vector expressing mitochondrially-targeted catalase (mCAT) (**Figures 2E–I, L**) which removes mitochondrial H_2_O_2_ (32). We also treated the cells with the mitochondrially targeted catalytic metalloporphyrin anti-oxidant, MnTBAP (**Figures 2J, K, M**) (33). Treatment with either MnTBAP or mCAT extinguished the 2-E+2-3a activated ROS product, confirming that the ROS was generated by the mitochondrion.

**Figure 3:**
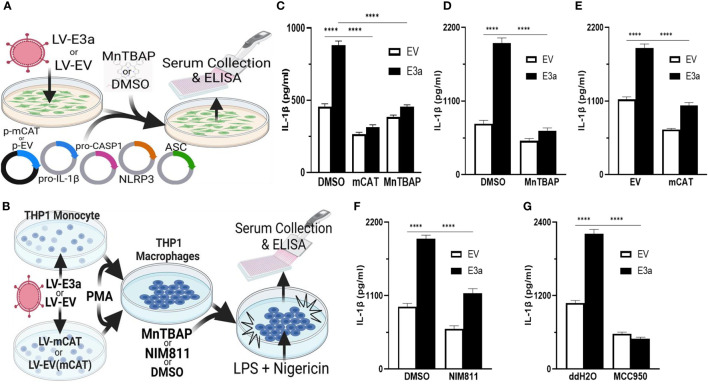
mROS and the mtPTP are required for activation of the NLRP3-I by the 2-E+2-3a viroporins. **(A, B)** Experimental design used to assess NLRP3-activated by IL-1β in cell-free supernatants quantified by ELISA. **(A)** 293T cells with an NLRP3-I reconstitution system (NLRP3, ASC, pro-CASP1, pro-IL-1β) and **(B)** THP1 differentiated into macrophages and primed with LPS + nigericin. **(C)** 293T cells transfected with LV-EV or LV-E3a were transfected with the NLRP3-I plasmids and p-mCAT or its control plasmid p-EV, or cultured in the presence or absence of 50 μM MnTBAP. **(D, F, G)** THP1 cells were transduced with LV-EV or LV-E3a, differentiated into macrophages, treated with LPS + nigericin, and treated with 5 μM MCC950, 100 μM MnTBAP or 10 μM NIM811, the supernatants analyzed for IL-1β by ELISA. **(E)** THP1 cells stably expressing LV-mCAT or control plasmid were infected with LV-EV or LV-E3a, differentiated into macrophages, and supernatant IL-1β levels determined *via* ELISA. Error bars represent SEM from 3 independent experiments; statistically significant data is indicated with asterisks (*).

2-E+2-3a induced mROS is involved in NLRP3-activation and IL-1β production. SARS-CoV-1, activation of the NLRP3-I and associated pathogenicity were observed for both viroporins 1-E+1-3a (11, 14, 34, 35). To determine if this is the case for SARS-CoV-2, we used two model systems to determine if 2-E+2-3a expression activates the NLRP3-I *via* the mitochondrion. In the first, we reconstituted the inflammasome in 293T cells by transforming with plasmids encoding the components of the NLRP3-I (**Figure 3A**) (25). In the second, we employed the human acute monocytic leukemia derived cell line, THP1, which can be converted to macrophages by treatment with phorbol ester (PMA) and the macrophages treated with LPS + nigericin (**Figure 3B**) (36). The reconstituted NLRP3-I 293T cells were transduced with the 2-E+2-3a expression vector (**Figure 3A**). The THP-1 cells were first transduced with the 2-E+2-3a expression vector and then treated with PMA and LPS + nigericin (**Figure 3B**). The expression of 2-E+2-3a in both cell systems, 293T (**Figure 3C**) and THP1 macrophages (**Figures 3D–F**) resulted in enhanced secretion of NLRP3-activated IL-1β in 293T-NLRP3-I (**Figure 3E**) and THP1 macrophages (**Figures 3D, E**).

We then confirmed that 2-E+2-3a expression activates the NLRP3-I and IL-1β secretion *via* increased mROS production. 293T cells expressing the inflammasome proteins (**Figure 3C**) and LPS-nigericin treated THP1 macrophages (**Figures 3D, E**) were treated with mitochondrially targeted antioxidants: transformation with mCAT or treatment with MnTBAP. Both mCAT expression and MnTBAP treatment impaired IL-1β secretion.

We then determined if mROS activation of the NLRP3-I was mediated by release of a mitochondrial component *via* the mtPTP, which has been conjectured but not proven. We treated 2-E+2-3a transduced THP1 macrophages with the specific mtPTP inhibitor N-methyl-4-isoleucine-cyclosporin (NIM811). NIM811 blocks the mtPTP by binding to cyclophilin D, analogous to cyclosporin A (CsA), but without calcineurin inactivation (37–39). NIM811 treatment suppressed the secretion of IL-1β following LPS + nigericin activation of the THP1 macrophages (**Figure 3F**) demonstrating for the first time the mtPTP is the route by which a mitochondrial factor, presumably mtDNA, reaches the NRLP3-I. Finally, to confirm that 2-E+2-3a secretion of IL-1β occurred through NRLP3-I, we treated THP1 macrophages with MCC950, a selective small molecule inhibitor that binds to the NACHT domain of NLRP3 (40). MCC950 blocked IL-1β secretion in 2-E+2-3a expressing THP1 macrophages, demonstrating that E3a secretion of IL-1β, was mainly through the activation of the NLRP3-I (**Figure 3G**).

**Figure 4:**
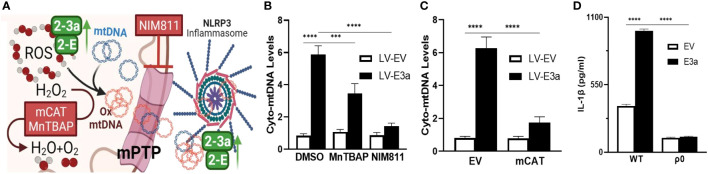
2-E+2-3a viroporins induced mROS is required for the release of mtDNA from the mitochondrion into the cytosol *via* the mtPTP. **(A)** Representative Figure. **(B, C)** 293T cells were transduced with LV-EV or LV-E3a. **(B)** 24 hrs post-transduction cells were treated with or without 50 uM MnTBAP or 10 μM NIM811. 10 hrs post-treatment, cells were lysed, cytosolic-enriched fractions collected, DNA isolated from the cytosolic-enriched fractions, and levels of cytosolic mtDNA determined by RT-PCR with a probe specific for *MT-ND5*. **(C)** 6 hrs post-transduction with LV-EV or LV-E3a, 293T cells were transfected with p-mCAT or its control plasmid p-EV. 24 hrs post-transfection, levels of cytosolic mtDNA were determined as described above. **(D)** 293T or 293T-ρ^0^ cells transduced with LV-EV or LV-E3a were transfected with the NLRP3-I plasmids the supernatants analyzed for IL-1β by ELISA. Error bars represent SEM from 3 independent experiments; statistically significant data is indicated with asterisks (*).

We next demonstrated that LV-E3a transduced 293T cells increased levels of cytosolic mtDNA *via* Real-Time PCR quantification of mitochondrial NADH dehydrogenase subunit 5 (*MT-ND5*) in cytosolic-enriched supernatants (**Figures 4A–C**). To confirm that 2-E+2-3a increased the levels of cytosolic mtDNA *via* efflux through the mtPTP, we treated 2-E+2-3a expressing 293T cells with NIM811, which extinguished the 2-E+2-3a elevation of cytosolic mtDNA. This confirms that mtPTP opening contributes to 2-E+2-3a elevation of cytosolic mtDNA (**Figure 4B**). To determine if mROS contributed to 2-E+2-3a release of mtDNA we transduced 293T cell expressing 2-E+2-3a cells with mCAT or treated the cells with MnTBAP. Both mitochondrial catalytic antioxidants impaired 2-E+2-3a elevation of cytosolic mtDNA (**Figures 4B, C**). Finally, to confirm that 2-E+2-3a activation of the NRLP3-I, was dependent on mtDNA we showed that secretion of the IL-1β was lost in 293T cells with a reconstituted inflammasome (**Figure 4D**) that had been cured of their resident mtDNA (ρ^0^ cells). The presence of mtDNA is thus an absolute requirement for 2-E+2-3a activation of the NRLP3-I.

Thus, we have demonstrated that co-expression of 2-E+2-3a enhances Ca^++^ leakage into the cytosol, increasing levels of cytosolic and mitochondrial Ca^++^. This 2-E+2-3a mediated increase in mitochondrial Ca^++^ increases mitochondrial NADH in association with increased mROS. mtDNA is then released into the cytosol *via* the mtPTP which activates the NLRP3-I stimulating the secretion of IL-1β. Increasing mitochondrial antioxidant defenses through treatment with the pharmacological mROS scavenger MnTBAP or genetic expression of mCAT blocks 2-E+2-3a induced activation of the NLRP3-I, which is absolutely dependent on the presence of mtDNA. Together these findings reveal that the mechanism by which 2-E+2-3a engage the NLRP3-I is *via* viroporin manipulation of mitochondrial physiology causing release of mtDNA to bind to the NLRP3-I.

## Discussion

A variety of mechanisms have been proposed to activate the NLRP3-I. These include interferon antagonism, organelle stress, mROS production, direct binding to inflammasome components (9, 10), and cellular expression of viroporins (16, 17). SARS-CoV-1/2 have been shown to agonizes the NLRP3-I by three mechanisms. First, through direct viral protein interaction with components or regulatory proteins of the NLRP3-I, *via* 2-N interaction with the NLRP3 receptor, and 1-3a interaction with CASP1 & TNF Receptor Associated Factor 3 (TRAF3) (13, 15, 36). Second, by increasing NF-κB-dependent target gene transcription of pro-inflammatory cytokines such as IL-18 and IL-1β through the 2-N, 2-S, 2-7a, 1-3a & 2-3a proteins (15, 36, 41, 42). Third, by distress or cellular damage caused by SARS-CoV-1/2 viroporins (11–13, 15, 43), which are transmembrane pore-forming proteins that localize to membranes and augment cell permeability to ions.

All human coronaviruses encode one or more viroporins that antagonize the NLRP3-I (44–47). SARS-CoV-1 encodes for three viroporins 1-E, 1-3a, and 1-8b. Several studies have shown that 1-E & 1-3a activate the NLRP3-I in various human cell lines transduced with the components of the NLRP3-I or LPS-primed macrophages and monocyte-derived macrophages (11, 13, 15). The 1-E viroporin has been reported to engage the NLRP3-I and elicit production of chemokines and cytokines through disrupting cellular Ca^++^ homeostasis (12, 14). Treatment of LPS-primed macrophages with the mROS scavenger MitoQ decreased 1-E or 1-3a activation of the NLRP3-I and secretion of IL-1β and co-expression of 1-E and 1-3a significantly increased levels of IL-1β secretion demonstrating a synergistic effect between the two viroporins (11).

SARS-CoV-2 encodes two viroporins 2-E & 2-3a, which share high sequence homology with their SARS-CoV-1 counterparts and function as cation-selective ion channels (19, 48). Both 1-3a & 2-3a stimulate NF-κB-dependent gene transcription and that 2-E increases the transcription of pro-inflammatory cytokines and chemokines, including IL-1β (13, 14, 42). However, the central role of the mitochondrion in viroporin activation of the NLRP3-I and the central role of mitochondrial Ca^++^, mROS, and mtDNA in viroporin-induced inflammation had not been determined. Our paper now demonstrates that the activation of the NLRP3-I *via* the 2-E and 2-3a proteins requires mitochondrial signaling *via* mitochondrial uptake of Ca^++^, increased NADH and mROS levels, the presence of mtDNA, and the release of mtDNA through the mtPTP. By inducing Ca^++^ release into the cytosol, 2-E+2-3a likely activates the tricarboxylic acid cycle dehydrogenases generating the excess NADH (21). The resulting excessive reducing equivalents likely then overloads the electron transport chain producing increased mROS. Presumably, the increased mROS damages the mtDNA and activates mtPTP opening. This increases cytosolic mtDNA, which can bind to and activate the NLRP3-I.

While our experiments did not confirm that the mtDNA released through the mtPTP was oxidized, other studies have shown that oxidized mtDNA is a ligand of NLRP3-I (10, 22). Thus, we complete the mitochondrial innate immune activation pathway by showing that the presence of mtDNA is required for activation of the NLRP3-I and that release of mtDNA is *via* the mtPTP.

Demonstration that SARS-CoV-2 activated the inflammasome *via* the mitochondria provides new approaches to mitigating the severity of the cytokine storm. Previous studies have indicated that generalized antioxidants such as N-acetyl cysteine (49), glutathione (50), and catalase (51) can reduce viral propagation and pathology. Our data extend these observations by identifying the sequence of events by which SARS-CoV-2 activates the NLRP3-I, thus demonstrating that mROS and the mtPTP are critical steps in NLRP3-I activation. Therefore more effective therapies may be obtained using mitochondrially targeted catalytic antioxidants such as MnTBAP (33), EUK-8, and EUK-134 (52) and also inhibitors of the mtPTP such as NIM811 (37, 38).

## Data availability statement

The original contributions presented in the study are included in the article/supplementary material. Further inquiries can be directed to the corresponding author.

## Author contributions

Conceptualization: JG, DW. Methodology: JG, PP, PS, DW, AA, DM. Literature and concept integration: JG, PP, DW, AA, DM. Formal Analysis: JG, PS, DW, AA, DM. Literature and concept integration: JG, P.P, DW, AA, DM, AA, DM, DW. Writing – Original Draft: JG. Investigation: JG, AA, DM, DW, TL, JH. Sample Collection: JG, T.L. Writing – Review & Editing: JG, DW, AA, DM, PS, TL. Visualization: JG, DW, AA, DM. Supervision: JG, P.P, DW, AA, DM. Funding Acquisition: DW. All authors contributed to the article and approved the submitted version.
